# Short- and long-term outcomes of single bare metal stent versus drug eluting stent in nondiabetic patients with a simple de novo lesion in the middle and large vessel

**DOI:** 10.1186/1479-5876-6-42

**Published:** 2008-08-06

**Authors:** Yue-jin Yang, Sheng Kang, Bo Xu, Ji-lin Chen, Shu-bin Qiao, Xue-wen Qin, Min Yao, Jue Chen, Yong-jian Wu, Hai-bo Liu, Jin-qing Yuan, Shi-jie You, Jian-jun Li, Jun Dai, Run-lin Gao

**Affiliations:** 1 Department of Cardiology, Cardiovascular Institute & Fu Wai Hospital, Chinese Academy of Medical Sciences & Peking Union Medical College, Bei Li Shi Rd 167, Beijing, 100037, PR China; 2 Department of Cardiology, Rui Jin Hospital, Shanghai Jiaotong University School of Medicine, Rui Jin 2nd Rd 197, Shanghai, 200025, PR China

## Abstract

**Objective:**

This study was aimed to investigate the short- and long-term outcomes of percutaneous coronary intervention (PCI) between single bare metal stent (BMS) and single drug eluting stent (DES) in nondiabetic patients with a simple de novo lesion in the middle and large vessel.

**Methods:**

Two hundred and thirty-five consecutive patients with a simple de novo lesion in the middle and large vessel were treated with BMS or DES in our hospital from Apr. 2004 to Dec. 2004.

The inclusion criteria: a simple de novo lesion in the middle and large vessel, stent diameter ≥ 3.0 mm, stent length ≤ 18 mm, the exclusion criteria: diabetes mellitus, left main trunk disease and left ventricular ejection fraction ≤ 30%. Of them, there were 150 patients in BMS group and 85 patients in DES group, and the rates of lost to follow up were 6.7% and 1.2% respectively.

**Results:**

BMS group had lower hypercholesteremia rate (22.0% vs 38.8%) and higher proportion of TIMI grade 0 (12% vs 1.2%) than DES group (all P < 0.05), but both groups had similar stent length (16.16 ± 2.81 mm vs 16.06 ± 2.46 mm) and stent diameter (3.85 ± 3.07 mm vs 3.19 ± 0.24 mm) after procedure, in-segment restenosis rate (0% vs 1.2%) and target lesion revascularization (TLR, 2.0% vs 2.4%) at 6-month follow-up (all P > 0.05). No difference was found in TLR (1.3% vs 1.2%, P = 1.00) and recurrent myocardial infarction (Re-MI) (0% vs 1.2%, P = 0.36), cardiac death (0.7% vs 1.2%, P = 1.00) between 1- and 3-year. So were TLR (6.0% vs 5.9%, P = 0.97), Re-MI (0% vs 2.4%, P = 0.06), cardiac death (2.0% vs 3.5%, P = 0.48) and major adverse cardiac events (MACE, 8.7% vs 10.6%, P = 0.63), cardiac death-free cumulative survival (98.7% vs 97.7%, P = 0.56), TLR-free cumulative survival (94.0% vs 94.1%, P = 0.98) and Re-MI-free cumulative survival (100% vs 97.7%, P = 0.06) at 3-year follow-up.

**Conclusion:**

The single BMS has similar efficacy and safety to single DES in nondiabetic patients with a simple de novo lesion in the middle and large vessel at short- and long-term follow-up.

## Background

Drug eluting stent (DES) has dramatically reduced restenosis risks compared with bare metal stent (BMS) and conventional balloon angioplasty [1-3]. Angiographic analysis found that the majority of DES restenosis were focal, localized, and bordered by segments with no angiographic evidence of neointima, while BMS restenosis tended to be diffuse or proliferative [4,5]. Thereby, DES has revolutionized revascularization therapy and is rapidly becoming the standard for percutaneous coronary intervention (PCI).

Although the incidence of late stent thrombosis is very low, DES may increase the risk for late events, especially associated with discontinuation of dual anti-platelet therapy [6,7]. Considering that the patients are difficult in anti-platelet compliance and more drug cost, we have to ask whether all lesions need DES and what specific lesion types are independent of DES therapy. Factors known to increase the risks of in-stent restenosis include smaller vessel diameter, prior restenosis, length of stented vessel, and diabetes mellitus [8,9]. However, few of study reported that a simple de novo lesion, for example, lesion type A/B1[10,11] in the middle and large vessel was treated with single BMS vs single DES at short- and long-term follow-up.

Thus we investigated the efficacy and safety of single BMS vs. single DES in nondiabetic patients with a simple de novo lesion in the middle and large vessel at 6-month, 1-year and 3-year follow-up in real world.

## Methods

### Study population

Demographic and procedural data were retrieved from a dedicated PCI database between Apr 2004 to Dec 2004 at Fu Wai hospital. Only a simple de novo lesion in single middle and large vessel, stent diameter ≥ 3.0 mm, stent length ≤ 18 mm were included. The unprotected left main disease ≥ 50% stenosis, left ventricular ejection fraction ≤ 30% and diabetic patients including definite diabetic patients, newly diagnosed patients and diet controlled patients were the major exclusion criteria. Finally, there were 150 patients in BMS group and 85 patients in DES group.

### Procedures and relevant medications

All patients were pretreated with aspirin and either ticlopidine or clopidogrel. A 300 mg loading dose of clopidogrel was administered before the procedure if patients were not pretreated. During the procedure, a bolus dose of unfractionated heparin (100 U/kg) was administered after femoral or radial artery sheath insertion, with repeat bolus given as needed to maintain activated cloting time between 250 to 300 seconds. The administration of glycoprotein IIb/IIIa inhibitors Tirofiban was left to the operator's discretion. The operators were free to use the BMS or DES that they considered best. BMS included Coroflex Delta, Driver, Express 2, micro-Driver, Multi-Link Mini Vision, Multi-Link Vision, Multi-Link Zeta, Mustang and Tecnic Carbostent, DES included Cypher, Cypher Select, Firebird and Taxus Express 2.

All patients kept on aspirin therapy (300 mg/day for 3 months and 100 mg/day in lifelong time). Ticlopidine (500 mg/day) or Clopidogrel (75 mg/day) was administered for 6 to 12 months after DES implantation or for 3 months in BMS group.

### Clinical definitions and follow-up

The clinical data were reviewed to obtain from a computerized database by specialized personnel at the cardiovascular interventional center in Fu Wai hospital. Risk factors for coronary artery disease that were tabulated included diabetes mellitus (only if treated medically), hypertension (only if treated medically), and hyperlipidemia (only if treated medically or if serum cholesterol was 240 mg/dl), but in this study we excluded diabetic patients. The diagnosis of acute myocardial infarction (AMI) during hospitalization and follow-up was based on the presence of new Q wave on electrocardiogram and/or elevation of creatine kinase MB to at least three times the upper limit of the normal range [12]. Simpsons method was used for LVEF measurement by the blind to two observers.

Quantitative coronary angiography analysis was made using a validated, edge detection system (MED CON QCA software). Lesion length was defined as the distance from the proximal to the distal shoulder of the lesion. The degree of stenosis before and after angioplasty was measured after intracoronary injection of nitrates in the view showing the most severe stenosis, and expressed as the minimum lumen diameter and the linear percent lumen diameter reduction, using the average diameter of the nearest proximal and distal normal segments as the reference. In-segment restenosis was defined as diameter stenosis ≥ 50% within a previously stented segment (5 mm proximal and distal to stent) using follow-up angiograms. A blood flow rate of grade 1 or higher according to the classification of the Thrombolysis in Myocardial Infarction (TIMI) trial.

Stent thrombosis was defined as occlusion of either vessel or thrombus within or adjacent to a previously successfully stented vessel from angiographic evidence or, in the absence of angiographic confirmation, either AMI in the distribution of the treated vessel or death not clearly attributable to other causes [13]. In-stent thrombosis was categorized according to the timing of the event into: acute thrombosis (within 24 hours after the procedure), subacute thrombosis (from postprocedure 1 to 30 days), late thrombosis (> 30 days and < 1 year) and very late thrombosis (≥ 1 year). Target lesion revascularization (TLR) was defined as any symptom driven coronary artery bypass graft or repeat PCI for restenosis or closure of the target lesion. MACE included recurrent myocardial infarction (Re-MI), cardiac death and TLR. Data for patients who did not have MACE were censored either at 3 years or at the last known time of follow-up. Data for patients who died before 3-year follow-up were censored at the time of death.

A patient's clinical status was assessed by outpatient interview or telephone conversation. All patients were asked to return for coronary angiography approximately six months after the procedure, or earlier if angina symptoms occurred. Telephone interviews or outpatient interview were repeated at twelve months and three years after the procedure. Relevant data were collected and entered into a computerized database by specialized personnel at the cardiovascular interventional center in Fu Wai hospital.

### Statistical analysis

All statistical analyses were performed with SPSS for Windows (version 10.0, Chicago). Continuous variables were described as mean ± SD, and categorical variables were reported as percentages or proportions. The comparisons of continuous variables were performed with unpaired t-tests (normal distribution) and nonparametric Mann-Whitney U test (skew distribution). The analysis of categorical variables was performed with Fisher's exact test and Chi-square test. Kaplan-Meier time-to-event estimates was used for the primary events at 1-year and 3-year of follow-up, which were compared with the log-rank test between BMS group and DES group. All reported P values were two-sided, and a P value < 0.05 was considered statistically significant.

## Results

Baseline clinical characteristics were shown in table 1. Compared to DES group, the patients in BMS group had lower hypercholesteremia rate (22.0% vs 38.8%, P = 0.006), but age, gender, other risk factors for coronary artery disease and left ventricular function were similar in the two groups (all P > 0.05).

**Table 1:** Baseline Clinical Characteristics

Characteristics	BMS group (N = 150 Pts)	DES group (N = 85 Pts)	P value
Age (years)	56.66 ± 11.72	58.26 ± 11.14	0.307
Male (%)	80.7	78.8	0.734
Hypercholesteremia (%)	22.0	38.8	0.006
Hypertension (%)	50.7	60.0	0.168
Family history of CAD (%)	3.3	3.5	0.937
Smoking (%)	37.3	38.8	0.821
Unstable angina history (%)	72.0	75.3	0.198
Acute myocardial infarction (%)	34.7	23.5	0.075
LVEF (%)	63.24 ± 23.23	68.43 ± 17.06	0.108

Note: Pts, patients; CAD, coronary artery disease; LVEF, left ventricular ejection fraction.

During procedure and in-hospital, BMS group had higher proportion of TIMI grade 0 than DES group (12.0% vs 1.2%, P < 0.05), but other variable including calcified lesion (%), lesion length (mm), stent diameter (mm), percentage of lumen stenosis (%), balloon predilatation (%), stent length (mm), post-dilatation (%), vessel dissection (%), postprocedural residual stenosis (%) and in-hospital outcomes did not significantly differ (all P > 0.05) in table 2. Despite that BMS had higher acute thrombosis rate than DES (3.3% vs 0%, P = 0.162), these patients recovered reperfusion after thrombolysis and intra-aortic balloon pump therapy, there were not in-hospital TLR and death in BMS group.

Repeat coronary angiography at 6-month follow-up showed similar acute and subacute thrombosis (%), late thrombosis (%), in-segment restenosis (%), TLR (%) and composite of cardiac death or Re-MI (%) in the two groups (all P > 0.05) in table 3.

**Table 2:** Angiographic characteristics and in-hospital outcomes

Characteristics	BMS group (N = 150 Pts)	DES group (N = 85 Pts)	P value
Calcified lesion (%)			0.282
None	79.9	70.6	
Slightness	15.4	18.8	
Moderation	3.4	8.2	
Severity	1.3	2.4	
Lesion length (mm)	12.60 ± 4.05	11.65 ± 3.09	0.062
Percentage of lumen stenosis (%)	87.82 ± 8.70	85.73 ± 8.48	0.075
TIMI grade			0.030
0	12.0	1.2	
1	3.3	2.4	
2	14.7	17.6	
3	70.0	78.8	
Balloon predilatation (%)	63.3	56.5	0.300
Stent length (mm)	16.16 ± 2.81	16.06 ± 2.46	0.782
Stent diameter (mm)	3.85 ± 3.07	3.19 ± 0.24	0.050
Post-dilatation (%)	15.4	25.6	0.060
Vessel dissection (%)	0	1.2	0.363
Acute thrombosis (%)	3.3	0	0.162
Postprocedural residual stenosis (%)	0.21 ± 1.15	0.29 ± 1.61	0.656
In-hospital TLR (%)	0	1.2	0.183
In-hospital cardiac death (%)	0	0	......

Note: Pts, patients; TLR, target lesion revascularization.

**Table 3:** The procedural characteristics and 6-month outcomes

Characteristics	BMS group (N = 150 Pts)	DES group (N = 85 Pts)	P value
Subacute thrombosis (%)	0	0	......
Late thrombosis (%)	0	2.4	0.129
In-segment restenosis (%)	0	1.2	0.258
Composite of cardiac death or Re-MI (%)	0	1.2	0.362
TLR (%)	2.0	2.4	1.000

Note: Pts, patients; Re-MI, recurrent myocardial infarction; TLR, target lesion revascularization.

The rates of lost to follow up at 3-year follow-up were 6.7% and 1.2% between BMS and DES group. The both groups had not significant differences in primary events including TLR (1.3% vs 1.2%, P = 1.00) and recurrent myocardial infarction (Re-MI) (0% vs 1.2%, P = 0.36) or cardiac death (0.7% vs 1.2%, P = 1.00) between 1- and 3-year, So were Re-MI (%), cardiac death (%), TLR (%) and MACE (%) at 1- and 3-year follow-up (all P > 0.05) in table 4.

**Table 4:** Clinical outcomes at 1-year and 3-year follow-up between DES group and BMS group

Events	1-year follow-up			3-year follow-up			Between 1-year and 3-year follow-up		
	
	BMS group (N = 150 Pts)	DES group (N = 85 Pts)	P	BMS group (N = 150 Pts)	DES group (N = 85 Pts)	P	BMS group (N = 150 Pts)	DES group (N = 85 Pts)	P
Re-MI (%)	0	1.2	0.36	0	2.4	0.06	0	1.2	0.36
Cardiac death (%)	1.3	2.4	0.62	2.0	3.5	0.48	0.7	1.2	1.00
TLR (%)	4.7	4.7	1.00	6.0	5.9	0.97	1.3	1.2	1.00
MACE (%)	6.0	7.1	0.75	8.7	10.6	0.63	2.7	3.5	0.71

Note: Pts, patients; Re-MI, recurrent myocardial infarction; TLR, target lesion revascularization; MACE, major adverse cardiac events.

**Figure 1:**
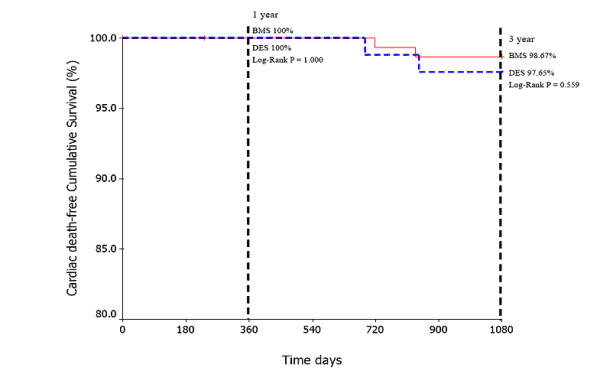
The Cardiac death-free cumulative survival between BMS group and DES group at 1-year and 3-year followup.

**Figure 2:**
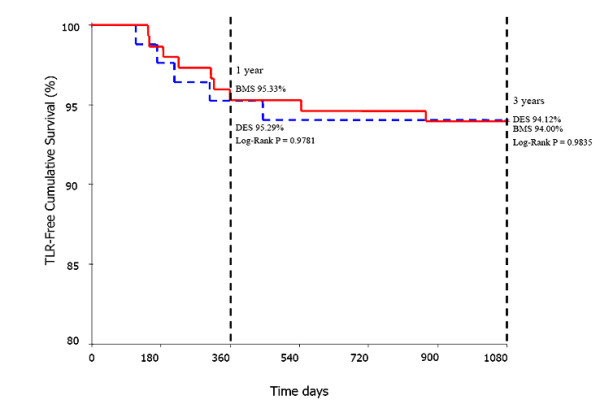
TLR-free cumulative survival between BMS group and DES group at 1-year and 3-year follow-up.

**Figure 3:**
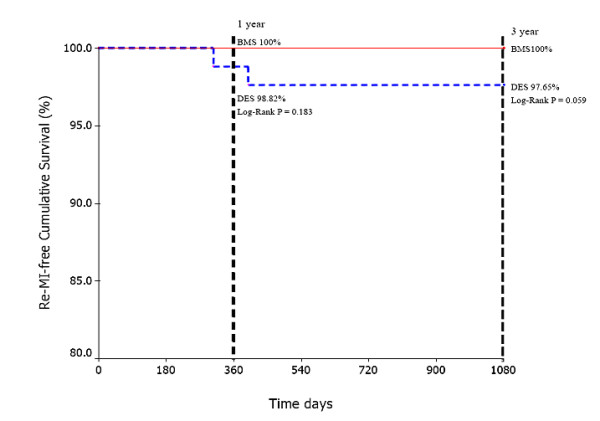
The Re-MI-free cumulative survival between BMS group and DES group at 1-year and 3-year follow-up.

The cumulative survival free of cardiac death in BMS group vs DES group was 100% vs 100% (Log rank P = 1.000) at 1-year and 98.67% vs 97.65% (Log rank P = 0.559) at 3-year follow-up (Fig. 1). Similarly, TLR-free cumulative survival between BMS group and DES group was 95.33% vs 95.29% (Log rank P = 0.978) at 1-year and 94.00% vs 94.12% (Log rank P = 0.984) at 3-year follow-up (Fig. 2). Noticeably, there was a trend towards a decrease of Re-MI-free cumulative survival in the DES group compared with the BMS group at 1-year (98.82% vs 100%, Log rank P = 0.183) and 3-year follow-up (97.65% vs 100%, Log rank P = 0.059) (Fig. 3).

## Discussion

This is the first study to investigate efficacy and safety of single BMS vs. single DES in nondiabetic patients with a simple de novo lesion in the middle and large vessel at 3-year follow-up in real world. The present study found that both DES group and BMS group had similar acute and subacute thrombosis (%), late thrombosis (%), in-segment restenosis (%), TLR (%), composite of cardiac death or Re-MI (%) at 6-month follow-up, so were Re-MI (%), cardiac death (%), TLR (%) and MACE (%) at 1- and 3-year follow-up in the two groups, furthermore, nonsignificant difference in the cardiac death-free and TLR-free cumulative survival rates except that there was a trend towards a decrease of Re-MI-free cumulative survival rate in DES group compared with BMS group at 1- and 3-year follow-up (all P > 0.05).

The previous study found that vessel diameter was an established predictor of angiographic outcome after catheter-based intervention, with a higher restenosis rate in smaller vessels [14]. Thereby, at the time of these pilot studies, sirolimus-eluting stents were only available in a 3.0 mm or 3.5 mm diameter, limiting treatment to relatively large vessels, these sirolimus-eluting stents showed 0% restenosis at 4-month [15], 6-month [16], and 12-month [17]. Later, a study demonstrated that the classic inverse relationship between vessel diameter and restenosis rate was seen in the BMS group but not in the sirolimus-eluting stent group [18], and vessel sizes of 2.5 – 3.5 mm were allowed in the subsequent randomized study with the sirolimus-coated Bx velocity balloon-expandable stent in the treatment of patients with de novo native coronary artery lesions (RAVEL) trial, yet lesions still had to be covered with one stent [19].

Currently, based on a lot of studies, people began to believe that the restenosis at the site of stent implantation seen in 15–60% of patients was dependent on various confounding factors, such as the presence or absence of diabetes mellitus, the size of the targeted coronary artery, the length of the coronary lesion, and the degree of vessel patency achieved by the intervention [20-25]. DES has been shown to reduce the risk of restenosis compared with BMS [1,19,25,26]. Despite that treatment of specific lesions types, especially in stent restenosis and distal stenosis of left main coronary, as well as diabetic patients, remains suboptimal with DES, whereas considering that DES practice including complex interventions is safe and associated with significant reductions in clinical driven repeat revascularization rates [27]. Moreover, DES also can effectively treat in-stent restenosis and saphenous vein graft restenosis [28-30], thus it appears to be the advent of transition from BMS to DES in routine PCI practice.

However, we do not disregard an important problem of DES, that is, thrombosis. Especially subacute in-stent thrombosis could occur more frequently with DES than with BMS and a prolonged anti-platelet regimen is mandatory [31]. In spite of the use of anti-platelet agents, stent thrombosis occurs in approximately 1% of patients, with an increased likelihood of occurrence in high-risk patients or complex lesion subset of patients [32,33]. According to the previous report, triple anti-platelet therapy (aspirin + clopidogrel + cilostazol) seemed to be more effective in preventing thrombotic complications after stenting than dual anti-platelet agent [34], but latterly a case report showed a patient with subacute stent thrombosis involving two different arteries simultaneously under the use of triple anti-platelet regimen [31]. Therefore, the promises of this potential panacea – DES, have been recently attenuated by the specter of late and very late stent thrombosis because of anti-platelet discontinuation [35-38]. However, the large-scale clinical trials and pool analysis demonstrated that the beneficial effect of DES on reducing the need for new revascularization compared with BMS extends to 4 years without evidence of a worse safety profile including thrombosis [39-42].

In our study, the specific lesion was choiced in the nondiabetic patients, and both BMS group and DES group had similar post-procedural outcomes including balloon predilatation (%), stent length (mm), stent diameter (mm), post-dilatation (%), vessel dissection (%) and postprocedural residual stenosis (%), finally we found that the both groups had similar acute and subacute thrombosis (%), late thrombosis (%), in-segment restenosis (%), TLR(%), composite of cardiac death or Re-MI (%) at 6-month follow-up and Re-MI (%), cardiac death (%), TLR (%) as well as MACE (%) at 1- and 3-year follow-up, so were the cardiac death-free and TLR-free cumulative survival rate, however, there was a trend towards a decrease of Re-MI-free cumulative survival rate in the DES group compared with the BMS group at 1- and 3-year follow-up (all P > 0.05), we presumed that the Re-MI might be associated with very late thrombosis. In view of less cost, short-term anti-platelet regimen, less thrombosis incidence, similar restenosis rate and TLR rate in BMS compared with DES, suggesting that BMS may has similar efficacy and superior safety compared with DES at 3-year follow up, thus the nondiabetic patients with a simple de novo lesion in the middle and large vessel seem to have other benefit from BMS instead of DES in real world.

### Limitation

Firstly, we investigated the non-diabetic patients with specific lesion in real world, the design of this trial was not randomized controlled trial (RCT), thereby the patients in BMS group had lower hypercholesteremia rate (22.0% vs 38.8%, P = 0.006) than DES group in baseline clinical characteristics, thereafter it is necessary for the RCT trials' investigation. Secondly, this is a small population and single medical center of investigation, thus it needs the large-scale trials to validate these findings. Thirdly, this study did not present very late thrombosis data, though it was few of incidence, the very late thrombosis should be investigated in future trials.

## Conclusion

The single BMS has similar efficacy and safety to single DES in non-diabetic patients with a simple de novo lesion in the middle and large vessel at short- and long-term follow-up.

## Authors' contributions

JLC, SBQ, XWQ, MY, JC, YJW, HBL, JiQY, SJY, JJL, JD and RLG carried out the relevant PCI procedures, BX participated in the design of the study, collected and managed the relevant data, YJY conceived of the study, participated in its design and coordination, and revised this manuscript. All authors read and approved the final manuscript.
